# Percutaneous sacral screw fixation alone sufficient for mildly displaced U-type sacral fractures with preserved osseous fixation pathways

**DOI:** 10.1007/s00590-023-03661-4

**Published:** 2023-10-24

**Authors:** Augustine M. Saiz, Patrick J. Kellam, Adeet Amin, Zachary Arambula, Abhi Rashiwala, Joshua L. Gary, Stephen J. Warner, Milton Routt, Jonathan G. Eastman

**Affiliations:** 1 Department of Orthopedic Surgery, The University of Texas Health Science Center at Houston, Houston, TX USA; 2 Department of Orthopaedic Surgery, The University of California Davis, Sacramento, CA USA; 3 Keck School of Medicine, Department of Orthopaedic Surgery, University of Southern California, Los Angeles, CA USA

**Keywords:** U-type sacral fracture, Lumbopelvic fixation, Percutaneous sacral screws, Pelvic ring injury

## Abstract

**Purpose:**

To describe U-type sacral fracture characteristics amenable to percutaneous sacral screw fixation.

**Methods:**

U-type sacral fractures were identified from a trauma registry at a level 1 trauma center from 2014 to 2020. Patient demographics, injury mechanism, fracture characteristics, and fixation construct were retrospectively retrieved. Associations between fracture pattern and surgical fixation were identified.

**Results:**

82 U-type sacral fractures were reviewed. Six treated with lumbopelvic fixation (LPF) and 76 were treated with percutaneous sacral screws (PSS) alone. Patients receiving LBF had greater sacral fracture displacement in coronal, sagittal, and axial planes compared to patients receiving PSS alone (*P* < 0.05), negating osseous fixation pathways. All patients went onto sacral union and there were no implant failures or unplanned reoperations for either group.

**Conclusion:**

If osseous fixation pathways are present, U-type sacral fractures can be successfully treated with percutaneous sacral screws. LPF may be indicated in more displaced fractures with loss of spinopelvic alignment. Both techniques for U-type sacral fractures result in reliable fixation and healing without reoperations.

## Introduction

U-type sacral fractures–a fracture morphology of bilateral vertical sacral ala fractures with a transverse fracture of a sacral body–are a subset of spinopelvic dissociation injuries [1]. These fractures occur both with high-energy and low-energy mechanisms, typically from a hyperflexion moment of the lower pelvis and lumbosacral junction [2, 3]. These fractures are inherently unstable and as such, nonoperative management is associated with poor outcomes [4, 5]. Surgery is the mainstay of treatment; however, a variety of techniques exist with the main options being percutaneous sacral screw fixation (iliosacral and trans-iliac trans-sacral) and lumbopelvic fixation. [6–10]

The literature has not defined clearly when percutaneous sacral screw fixation versus lumbopelvic fixation is indicated in these injuries. The benefit of percutaneous sacral screw fixation in isolation is the low surgical morbidity and can afford immediate weight bearing although screws alone are not as biomechanically robust as lumbopelvic fixation [11–13]. However, lumbopelvic fixation is often associated with longer procedures, more soft tissue risk, and greater surgical morbidity but provide the most stable construct, facilitate reduction of kyphotic U-type fractures, and allow immediate weight bearing. [13–15]

The goal of this study was to identify U-type fracture patterns and describe their treatment based on fracture patterns. The study aims to assist surgeons in determining which fractures can be treated with percutaneous sacral screw fixation and those fractures that necessitate lumbopelvic fixation.

## Methods

Adult patients (> 18 years old and skeletally mature) with U-type sacral fractures treated surgically at a level 1 trauma center from 2014–2020 were identified retrospectively from a prospective trauma registry database. U-type sacral fractures were identified based on initial diagnosis reported followed by review of the imaging, both radiographs and computed tomography (CT), to confirm. In addition to U-type sacral fracture presence, involvement of the anterior ring was also recorded. Patients with prior pelvic or spine injury stabilized with implants were excluded.

We recorded the mechanism of injury to account for low- and high-energy level injuries as well as maximal anterior-to-posterior and medial-to-lateral translational fracture displacement on the axial CT and the amount of kyphosis on the sagittal CT. We made note if osseous fixation pathways for 7.0 mm sacral screws were intact or compromised on the injury CT using the previously described “drive through” measurement [18], accounting for sacral dysmorphism if present. Surgical technique, percutaneous sacral screws or lumbopelvic fixation, was recorded. Number and position of sacral screws were recorded. Patients were followed for at least 6 months and presence of union was recorded. Postoperative complications of nonunion, implant failure, or unplanned reoperations were noted.

Statistical analyses were performed using GraphPad (San Diego, CA). Mean values are reported with a standard deviation. Paired Student t-tests using nonparametric parameters and χ2 tests were used for comparison of mean values with statistical significance set at *p* < 0.05.

## Results

A total of 82 patients with U-type sacral fractures were identified. Average patient age was 65 years old (19–94; 83% female) and 33% had another concomitant orthopedic injury. Average follow-up was 23.5 months (6–40). The most common mechanism of injury was ground level fall (78%) followed by motor vehicle collision (10%) (Table 1). On injury imaging, 54 patients had an anterior pelvic ring fracture (ramus fracture) in addition to the sacral U-type fracture, with 37 of these patients having unilateral anterior ring fracture and 17 patients with bilateral anterior ring fractures. There were no patients with pubic symphyseal disruption. Osseous fixation pathways for percutaneous sacral screws were intact in 76 of the patients on initial injury imaging. There were 4 patients with sacral dysmorphism among the group. We defined sacra without a trans-iliac trans-sacral screw path in the upper sacral segment dysmorphic for a functional definition.

**Table 1:** Injury characteristics of the U-type sacral fractures

Descriptive characteristics	Number	%
U-type sacral fracture with anterior ring injury	54	65.8
Unilateral	37	45.1
Bilateral	17	20.7
Energy level of injury		
High	8	10
Low	74	90
Sacral dysmorphism	4	7.3
Intact osseous fixation pathways	76	92.7

Lumbopelvic fixation was used in 6 patients with the other 76 being treated with percutaneous sacral screws only (Table 2). Osseous fixation pathways were present on the injury CT for all patients treated with percutaneous screws only, whereas osseous fixation pathways were compromised in the lumbopelvic group (Table 3). As a result, patients treated with percutaneous sacral screws only tended to have multiple screws in the upper sacral segment compared to patients treated with lumbopelvic fixation. Most commonly, patients treated with percutaneous sacral screws had two trans-iliac trans-sacral screws placed in the upper sacral segment; if dysmorphism was present, often patients had two oblique style iliosacral screws in the upper segment ± trans-iliac trans-sacral screw in S2 corridor if the transverse fracture pattern was amenable. In both groups, sacral dysmorphism was equally present.

**Table 2:** Treatment of the U-type sacral fractures

Descriptive characteristics	Number	%
Percutaneous screws only	76	92.7
Trans-iliac trans-sacral screws in upper segment	74	97.4 (74/76)
Iliosacral screws in upper segment	2	2.6 (2/76)
1 screw	3	3.9
2 screws	62	81.6
> 2 screws	11	14.5
Lumbopelvic fixation	6	7.3
Additional percutaneous screw	6	100
Anterior ring fixation	50	92.6% (50/54)

**Table 3:** Characteristics of lumbopelvic fixation and percutaneous screw only fixation

Descriptive characteristics	LBF (*n*, %)	PSS (*n*, %)	*p*
High-energy injury mechanism	6 (100)	10 (13)	< 0.001
Sacral dysmorphism	1 (17)	3 (4)	0.17
Intact osseous fixation pathways	2 (33)	76 (100)	< 0.001
Multiple screws in upper sacral segment	0 (0)	73 (96)	< 0.001
Anterior ring fixation	4 (67)	50 (66)	0.51
Maximal translational displacement on CT (mm)	18.9 (± 8.1)	7.4 (± 5.6)	< 0.001
Kyphotic angulation (degrees)	30.7 (± 10.7)	14.2 (± 8.6)	0.007
Surgery-related complications	0 (0/6)	6 (8)	0.46

*LBF* lumbopelvic fixation, *PSS* percutaneous screw only fixation, *n*–number

Patients with high-energy mechanisms were more likely to have lumbopelvic fixation compared to percutaneous sacral screw fixation. As such, fractures treated with lumbopelvic fixation tended to be more displaced and more angulated compared to those fractures treated with percutaneous screws only. Anterior ring fixation was commonly done to address anterior ring fractures in both groups. All patients were weight bearing as tolerated postoperatively. There were no differences in postoperative union in either group or surgical complications.

## Discussion

Sacral U-type fractures vary on a spectrum from low-energy to high-energy injuries. As such, no standard treatment algorithm exists for how to best manage these fractures. However, two mainstay surgical treatment strategies have been lumbopelvic fixation and percutaneous sacral screws [5, 12, 13, 16]. Our study provides further insight to the fracture patterns that predispose particular U-type sacral fractures to one or the other surgical modality.

Selection of fixation construct in our cohort of U-type sacral fractures depended primarily on fracture pattern. Fractures that preserved osseous fixation pathways, especially for two trans-iliac trans-sacral screws in the upper sacral segment and had less initial translational and kyphotic angulation on injury imaging, were more likely to be treated with percutaneous sacral screws alone. The more displaced fractures with loss of osseous fixation pathways were more high-energy injuries that required lumbopelvic fixation.

These findings are in concordance with previous literature. Pulley et. al. recently reported on a case series of 114 patients with low-energy pelvic ring injuries, 19 of which had U-type sacral fractures [10]. All were treated with percutaneous screws only, with 13 having two trans-iliac trans-sacral screws placed in the upper sacral segment. Wright et. al. recently provided a technique paper describing fixation of osteoporotic U-type fractures and advocated for multiple screws in the upper sacral segment if able in order to maximize implant purchase and stability [17]. Furthermore, the study describes the use of lumbopelvic fixation in fractures with severe kyphosis.

**Fig. 1:**
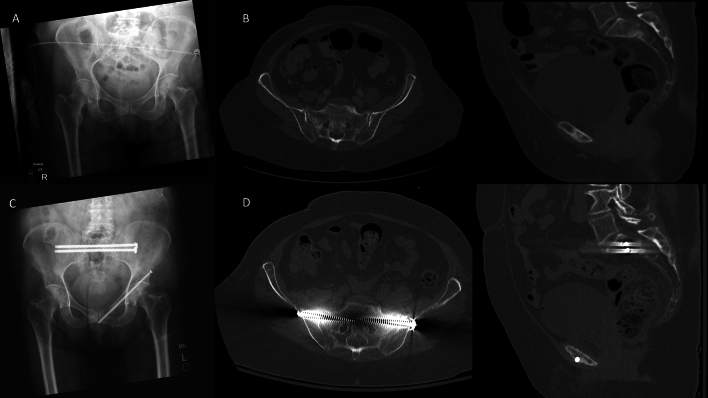
U-type sacral fracture with minimal displacement treated with percutaneous sacral screws. **A** Injury radiographs. **B** On axial and sagittal CT cuts, minimal displacement is noted, and osseous fixation pathways preserved for multiple trans-iliac trans-sacral screws in the upper sacral pathway. **C** Postop radiographs. **D** Postoperative CT

**Fig. 2:**
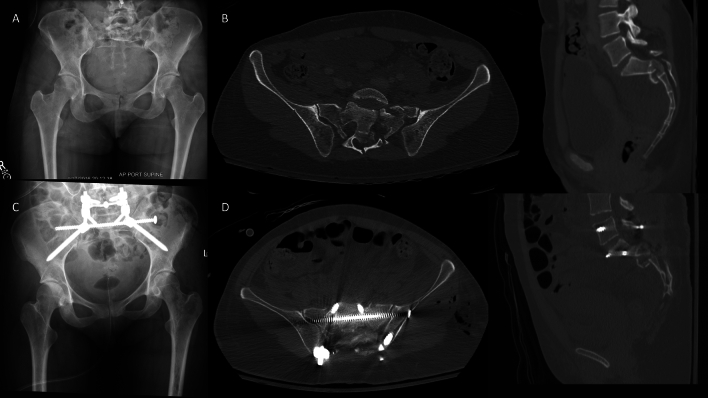
U-type sacral fracture with significant displacement treated with lumbopelvic fixation. **A** Injury radiographs. **B** On axial and sagittal CT cuts, minimal displacement is noted, and osseous fixation pathways preserved for multiple trans-iliac trans-sacral screws in the upper sacral pathway. **C** Postop radiographs. **D** Postoperative CT

Our study suggests that in fracture patterns where osseous fixation pathways are preserved, correlating with less degree of displacement, that percutaneous screw fixation with multiple screws (favorably trans-iliac trans-sacral screws), is safe and effective in healing these fractures without residual displacement (Fig. 1). This requires scrutiny of the injury CT to evaluate the osseous fixation pathways present [18, 19]. Whereas, when a reduction is required to restore osseous fixation pathways and/or displacement is significant, lumbopelvic fixation is indicated (Fig. 2). Although reduction of these injuries, typically the kyphosis, can restore osseous fixation pathways, the biomechanical advantages of lumbopelvic fixation can ensure stability. [14]

There are limitations to our study beyond those inherent to a retrospective analysis. First, due to the sample size, the study may be underpowered and confidence in significant differences may be undermined. Furthermore, our study lacks clinical data, especially time from injury to surgery, and patient reported outcomes. For example, although patients were allowed to be weight bear as tolerated, we do not have accurate data regarding whether patients were immediately actually weight bearing or not, with or without assistive device. Additionally, our study excludes patients with sacral insufficiency fractures not seen on radiographs or CT as we did not review MRIs. Finally, this cohort is heterogeneous in terms of patient demographics, type and timing of surgery, mechanism of injury, fracture patterns, and sacral dysmorphisms presence resulting in lack of matched-patient control analysis. Nonetheless, this study does provide a general pattern of treatment strategy based on fracture pattern alone. Further study on the topic of U-type sacral fractures would benefit from subcategorizing and matched analysis of different surgical techniques accounting for patient reported outcomes.

## Conclusion

U-type sacral fractures represent a spectrum of injury, from relatively low-energy fractures with mild displacement to high-energy injuries with significant fracture displacement. A determining factor in deciding between percutaneous sacral screws versus lumbopelvic fixation is fracture displacement related to preservation of osseous fixation pathways. Most fractures with maintained spinopelvic alignment along with osseous fixation pathways for multiple screws can be treated with percutaneous sacral screws alone. Lumbopelvic fixation may be indicated in more displaced fractures with loss of spinopelvic alignment. Both techniques for U-type sacral fractures result in reliable fixation and healing.
